# Beyond Eosinophilic Esophagitis: Eosinophils in Gastrointestinal Disease—New Insights, “New” Diseases

**DOI:** 10.1093/jcag/gwad046

**Published:** 2023-11-24

**Authors:** Nicholas J Talley, Grace L Burns, Emily C Hoedt, Kerith Duncanson, Simon Keely

**Affiliations:** University of Newcastle, University Drive, Callaghan NSW, Australia; NHMRC Centre of Research Excellence in Digestive Health, Locked Bag 1000, New Lambton NSW 2305, Australia; University of Newcastle, University Drive, Callaghan NSW, Australia; NHMRC Centre of Research Excellence in Digestive Health, Locked Bag 1000, New Lambton NSW 2305, Australia; University of Newcastle, University Drive, Callaghan NSW, Australia; NHMRC Centre of Research Excellence in Digestive Health, Locked Bag 1000, New Lambton NSW 2305, Australia; University of Newcastle, University Drive, Callaghan NSW, Australia; NHMRC Centre of Research Excellence in Digestive Health, Locked Bag 1000, New Lambton NSW 2305, Australia; University of Newcastle, University Drive, Callaghan NSW, Australia; NHMRC Centre of Research Excellence in Digestive Health, Locked Bag 1000, New Lambton NSW 2305, Australia

**Keywords:** Eosinophils, mast cells, functional dyspepsia, microbiome, food antigens

## Abstract

Functional dyspepsia (FD) is a highly prevalent disorder. Upper endoscopy is normal, and according to the Rome IV criteria, there is no established pathology. Data accumulated over the last 15 years has challenged the notion FD is free of relevant pathology, and in particular, increased duodenal eosinophils have been observed. Intestinal eosinophils play important roles in microbial defence, immune regulation, tissue regeneration and remodelling, and maintaining tissue homeostasis and metabolism; degranulation of eosinophils releases toxic granule products (e.g., major basic protein, eosinophil-derived neurotoxin) which can damage nerves. Normal cut-offs for eosinophil infiltration into the duodenum histologically are less than five eosinophils per high power field (<25 per five high power fields). In clinical practice there is evidence that pathologically increased intestinal eosinophils may often be overlooked. In a meta-analysis duodenal eosinophils were significantly increased in FD although there was substantial heterogeneity; degranulation of duodenal eosinophils was also significantly higher in FD without significant heterogeneity. In addition, increased duodenal permeability, systemic immune activation, and an altered mucosa-associated duodenal microbiome have been identified that may help explain why symptoms arise, often occur after food with exposure to food antigens, and typically fluctuate. Several potentially reversible risk factors for FD have now been identified. We evaluate the current evidence linking duodenal microinflammation and immune activation with FD and disorders of gut–brain interactions that overlap with FD. We propose a two-hit disease model for eosinophilic functional dyspepsia (EoFD) with management implications.

## Introduction

Disorders of gut–brain interactions (DGBIs) are highly prevalent, unexplained conditions that can markedly impact on quality of life. After irritable bowel syndrome (IBS), the most recognized DGBI globally is functional dyspepsia (FD), characterized by chronic early satiety and/or postprandial fullness (termed postprandial distress syndrome) and/or epigastric pain or burning (termed epigastric pain syndrome).^1^ Traditionally, DGBIs have been defined as disorders that have no established pathology.^2^ However, data accumulated over the last 15 years has challenged the notion FD is free of relevant pathology, and in particular increased duodenal eosinophils and intestinal immune activation have been observed in this disorder.^3^ In this review we will summarize the evidence linking FD with eosinophilic gastrointestinal diseases (EGIDs), which is termed eosinophilic functional dyspepsia (EoFD).

## Eosinophil biology

Eosinophils are granulocytes mostly associated with innate immune responses to large parasites such as helminths, or cytotoxic effector cells in host allergic responses.^4^ Under normal conditions, eosinophil numbers in the peripheral blood are relatively low, representing approximately 2–5% of circulating leukocytes. However, eosinophils are enriched in the mucosal tissues that interface with the host environment, including the skin, uterus, airways, and the intestinal tract, which has the largest reservoir of eosinophils in the body.^5^ This enriched mucosal distribution contributes to the conventional theory that eosinophils evolved with host–parasite interactions.^6^

While the field of eosinophil biology developed with a focus on parasitic immunity and allergy, for which the eosinophil/T helper 2 (Th2) signalling axis is crucial,^7^ over a decade ago the Local immunity and/or remodeling/repair (LIAR) hypothesis was proposed^8^ implicating eosinophils in a broader range of biological processes, including microbial defence,^9^ immune regulation,^10^ tissue regeneration and remodelling,^11^ and maintaining tissue homeostasis and metabolism,^12^ suggesting that eosinophils play a more nuanced and complicated role in innate immunity than simple effectors of host defence.

Within the GI tract, eosinophils play a vital role in the maintenance of tissue homeostasis, particularly in the small intestine, where they are enriched in the duodenum and jejunum.^13^ Morphology studies in mice show that eosinophils are distributed throughout the lamina propria villus and crypt areas and can be present in close proximity to stromal cells, glial cells, and neuronal axons, which may indicate a role in gut–brain signalling.^14^ On the other hand, eosinophils were found to be absent in the muscularis and their distribution in the tissue may be regulated by proximity to the mucosal microbiome. Indeed, germ-free (GF) mice exhibit increased mucosal eosinophil numbers compared to those housed in specific pathogen-free conditions.^15^ Reconstitution of the microbiota in GF mice leads to increased eosinophil turnover which may be a result of increased epithelial IL-33 release.^14^ This suggests that resident intestinal eosinophils may display a phenotype regulated by the microbiota and intestinal environment, and this is supported by transcriptomic analysis that shows that small intestinal eosinophils exhibit changes in the expression of more than 13,000 genes when compared to bone marrow-derived eosinophils.^16^

Single-cell RNA sequencing (scRNA-seq) in mice has defined two distinct eosinophil lineages: a basal population (SiglecF^+^ CD80^−^ PDL1^−^) and an active population (SiglecF^+^ CD80^+^ PDL1^+^) specific to the intestine.^17^ Intestinal eosinophils respond to the tissue microenvironment which includes exposure to foreign antigens, the commensal microbiota, and potential pathogens. Small intestinal eosinophils also play a role in maintaining regulatory T cell (Treg) balance and as such are likely to be involved in the maintenance of oral tolerance, preventing immune activation against harmless foreign antigens.^18^

Eosinophils possess an array of receptors and growth factors involved in wound repair. The expression of histamine receptors^19,20^ allows them to sense and partake in the resolution phase of inflammation. Eosinophil chemoattractants are regulated by tissue regenerative triggers such as hypoxia^21^ and epithelial damage signals to adjacent eosinophils secretion of transforming growth factors (TGF)^22–24^ which promotes epithelial proliferation and, along with matrix metalloproteinase (MMP-9),^25,26^ extracellular matrix deposition and modification, while eosinophil secretion of fibroblast growth factors (FGF) and vascular-endothelial growth factors (VEGF)^22,27^ drive remodelling during tissue repair.

Activated eosinophils damage tissues by releasing toxic granule products (e.g., major basic protein, eosinophil-derived neurotoxin) which can destroy nerves.^28^ They also release lipid mediators (e.g., sulfidopeptide leukotrienes, platelet-activating factor) that can mediate smooth muscle contraction and alter motility. Degranulation of eosinophils in tissue traps may lead to an apparent eosinophil absence that can be misleading.^28^

## Eosinophil numbers in the gastroduodenum: What is and is not normal?

Eosinophils are normally found in both the stomach and duodenum but not the oesophagus. Normal cut-offs for eosinophil infiltration into the stomach and duodenum histologically typically are less than five eosinophils per high power field. Notably the high-power field size varies by microscope which needs to be considered when comparing studies.^29,30^

We conducted a true random population-based study in Sweden and counted eosinophils in subjects without any GI symptoms or known GI disease and who were otherwise healthy (“true normal”).^31^ Over 80% of randomly identified subjects invited agreed to undertake an oesophagogastroduodenoscopy and the sample included was very similar to the background population with no evidence of selection bias. The normal mean duodenal eosinophil count in five high power fields in the duodenal bulb and second portion was 18.4 (SD, ±10.9) and 18.6 (SD, ±10.5) in controls, respectively (i.e., 4–6 per high power field). Lower eosinophil counts were identified in the stomach in health (median <11 eosinophils per five high power fields).^31,32^ Similar normal duodenal eosinophil cut-offs were reported in a meta-analysis of paediatric patients who did not have a disorder of gut–brain interactions and were apparently disease free.^33^

In eosinophilic gastritis (EoG) and eosinophilic duodenitis (EoD), varying thresholds have been applied to diagnose disease, ranging from 20 to more than 50 eosinophils per high power field (hpf) in 3–5 fields (i.e., 4–10-fold increased eosinophil numbers above normal).^30^ Some have suggested eosinophils in the range of 20–50 per high power field in the duodenum may be borderline normal and very high eosinophil tissue values (>52 eosinophils/hpf) could be more likely to be associated with evidence of histologic or endoscopic damage.^34^ However, there is no compelling evidence these higher eosinophil counts correlate with more duodenal tissue damage.

In a retrospective study of children and adults whose endoscopy revealed no evidence of GI or systemic disease (*n* = 123), the mean and peak eosinophil counts in gastric biopsy specimens were 3.8 ± 3.6 eosinophils/hpf and 5.8 ± 5.0 eosinophils/hpf, respectively, while the mean and peak eosinophil counts in duodenal biopsy specimens were 14.6 ± 8.9 eosinophils/hpf and 19.5 ± 11.0 eosinophils/hpf, respectively.^35^ In the same study patients with an established EGID were enrolled (*n* = 52), and a mean count of 20 eosinophils/hpf in gastric biopsy specimens or 30 eosinophils/hpf in duodenal biopsy specimens identified patients with EGIDs with high specificity.^35^

EGIDs in clinical practice may often be overlooked and misdiagnosed. For instance, in a large pathology database, Genta et al. found that a clinical suspicion of eosinophilic gastritis or duodenitis was rarely reported (0.001% of 1,438,206 gastric biopsies and confirmed in 11.5% of them; 0.02% of 675,519 patients with duodenal biopsies and confirmed in 8.0%).^36^ Furthermore, of 31 US community pathologists assessed, most neither reported increased gastric or duodenal eosinophilia, nor diagnosed obvious EoG and EoD.^37^ However, the yield did improve when pathologists were provided histories suggestive of an eosinophilic gut disorder.^37^ In a study of 88 patients in secondary GI care with moderate–severe GI symptoms consistent with DGBIs, 72 met the histological criteria for EoD and/or EoG yet had not previously been diagnosed as EGID.^38^ Notably increased gastroduodenal eosinophilia was patchy and required examination of multiple biopsies; on average three per eight gastric biopsies and two per four duodenal biopsies per subject met pre-specified pathological thresholds. It was estimated four duodenal biopsies (and eight gastric) are required to capture 100% of patients with eosinophilic duodenitis or gastritis.^38^

While there is agreement that high tissue eosinophil counts, especially observed in sheets, are abnormal, other evidence suggests modestly increased eosinophil values in the duodenum (above 25 in 5 hpf) are associated with symptoms and increased intestinal permeability, consistent with disease.^31,32,39^ Pathologically high eosinophil counts can be found in a minority of healthy controls. In a US study in secondary GI care where we included virtually asymptomatic healthy controls, of 33 evaluable subjects, two (6%) met the strict histologic criteria for eosinophilic gastritis or duodenitis.^40^

## Eosinophilic gastritis and eosinophilic duodenitis: increasing in prevalence

Eosinophilic gastrointestinal diseases (EGIDs) distal to the oesophagus have been considered rare disorders but may be underdiagnosed and have been increasingly recognized when standardized pathology protocols have been applied in research studies. The catch all term eosinophilic gastroenteritis is no longer recommended.^41^ After eosinophilic esophagitis (EoE), EoG and EoD are the EGIDs most frequently diagnosed. Mucosal disease is most often recognized, but the serosa is very rarely involved leading to eosinophilic ascites, while muscle layer disease may present with symptoms of intestinal obstruction.^42^

EGIDs have been thought to be rare in clinical practice,^43^ but in a meta-analysis of 10 studies comprising 13,377 patients, the prevalence of non-EoE EGIDs in patients with GI symptoms was 1.9% (95% confidence interval: 0.6–3.9) although there was high heterogeneity.^44^ In a retrospective US study where data were collected from six centres from 2005 to 2016 in 373 subjects (317 children and 56 adults), rates of diagnosis of both EoG and EoD have increased over time.^45^ The incidence of EGIDs like other atopic diseases may also be increasing, although compelling data are unavailable.^45^

EoG and EoD occur in females as often as in males and may be misdiagnosed as a DGBI as the symptom presentation of mucosal disease (abdominal pain, early satiety, bloating, diarrhoea, and nausea) is often identical.^42,46^ Therefore, some DGBI cases might be explained by the presence of an unrecognized EGID (Table 1) and there is increasing evidence suggesting this is likely.

**Table 1.** Classic eosinophilic gastritis/enteritis (EGID) vs. eosinophilic duodenitis in functional dyspepsia.

Eosinophilic gastritis/enteritis	Eosinophilic duodenitis and FD
• Rare	• Common, may be missed
• Male = female	• Female preponderance
• Dense eosinophilia, may be in sheets	• Subtle eosinophilia—must count 5 HPF
• Strong atopic background (50–70%)	• Atopy increased but not in majority
• Pain, nausea, vomiting, early satiety, diarrhoea, malabsorption may occur	• Pain, early satiety, postprandial fullness, overlap with IBS
• Can be symptom/histologic disconnect	• Can be symptom/histologic disconnect
• Mucosal. Rarely muscular layer or serosal	• Mucosal layer only (as far as we know)
• Peripheral eosinophilia (>500/μL) common	• Peripheral eosinophilia (>500/μL) rare
• May respond to an exclusion diet	• May response to an exclusion diet
• May be steroid responsive	• Role of steroids is unclear (1 RCT)

## Eosinophilic duodenitis and disorders of gut–brain interactions

**Fig. 1.**
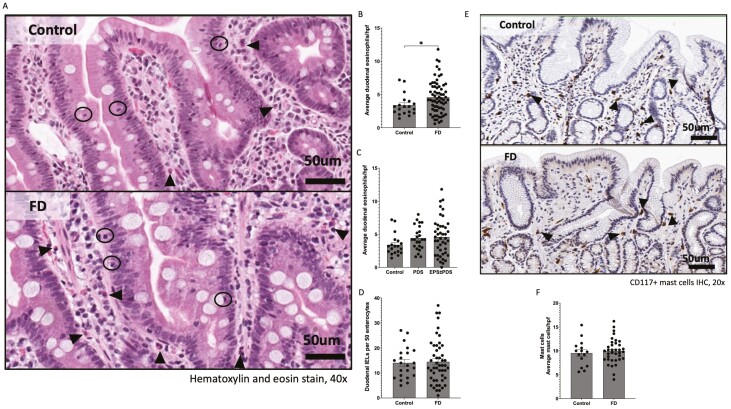
Increased duodenal eosinophils in functional dyspepsia (FD) versus healthy controls. Biopsies were collected from the second portion of the duodenum (D2) of FD patients and controls and stained with (A) haematoxylin and eosin (40× magnification, arrows indicate eosinophils, circles indicate intra-epithelial lymphocytes (IELs) scale bar = 50μm). (B) Average duodenal eosinophil counts per five high powered fields were examined between controls and FD. (C) Average duodenal eosinophil counts within the FD subtypes. (D) Duodenal intra-epithelial lymphocytes (IELs) were counted across groups using H&E-stained sections and were not increased. (E) Immunohistochemical staining of CD117+ mast cells performed (20× magnification, arrows indicate CD117+ cells, scale bar = 50μm). (F) Mast cells were counted based on CD117+ positively stained cells (brown) in all groups. Mast cells based on immunohistochemical staining of CD117+ were not increased. *n* = 15–22 for controls, *n* = 38–67 for FD, *n* = 24 postprandial distress syndrome (PDS), *n* = 43 epigastric pain syndrome (EPS) ± PDS. Data presented as mean ± SEM. From Burns G. et al. Front Immunol. 6, no. 13 (January 2023): 1051632. doi: 10.3389/fimmu.2022.1051632.

There are now compelling data showing that increased duodenal eosinophils are associated with some common unexplained upper GI disorders classified as DGBIs (fig 1). We conducted the first nested case–control study to evaluate duodenal pathology in FD community subjects and normal controls randomly selected from the population. Because duodenal visceral hypersensitivity has been documented in FD, EoD can present with symptoms indistinguishable from FD, and immune activation with circulating small intestinal homing T-cells are present in IBS which strongly overlaps with FD,^47–49^ We hypothesized that there would be increased duodenal eosinophils in subjects with FD.^31^ In our random sample of 1001 adult Swedish people, 16% were diagnosed with FD by Rome criteria (including a normal esophagogastroduodenoscopy). The odds ratio for FD in those with high duodenal (D2) eosinophil counts, adjusting for age, sex, and *Helicobacter pylori* status was sevenfold increased (vs. controls). We observed a significantly higher number of eosinophil clusters in the duodenum in FD. By immunostaining with major basic protein antibody, increased eosinophil degranulation was also found in FD. We did not find increased gastric eosinophils in FD. Consistent with these pathological observations, we later confirmed that increased small intestinal homing T-cells are present in FD supporting the concept of ongoing active intestinal inflammation in this DGBI.^50^ We have also shown both FD and IBS are associated with atopic disease,^51,52^ supporting the hypothesis immune activation underlies a major subset of DGBIs.^53^

Subsequently, we and others independently confirmed the initial findings. In a meta-analysis of 22 case-control studies comprising 1,108 patients with FD and 893 controls, duodenal eosinophils were significantly increased in FD but there was substantial heterogeneity.^54^ Degranulation of duodenal eosinophils was also significantly higher in FD without significant heterogeneity. Notably, postinfectious FD in a sub-analysis was associated with increased duodenal eosinophils compared with controls.^54^

A provocative study enrolled patients from secondary GI care practices across the United States with moderate to severe chronic GI symptoms suggestive of an EGID, including abdominal pain, nausea, early satiety, and diarrhoea. The majority of these patients (95%) had been already fully investigated and given a diagnostic label of IBS, gastroesophageal reflux disease (GERD) or, least often, FD. Based on the symptoms recorded, it seems likely most of these patients would have met Rome IV criteria for FD and/or diarrhoea-predominant IBS but this was not specifically evaluated, a limitation of the study. Overall, 405 patients met the moderate–severe symptom criteria and underwent endoscopy with multiple gastric (*n* = 8) and duodenal biopsies (*n* = 4). Strikingly, 181 (45%) met histologic criteria for EoD alone (75%) or EoG alone, or both (defined as and ≥30 eos/hpf in ≥3 duodenal hpfs, and ≥30 eosinophils per hpf in ≥5 gastric hpfs). These data support the hypothesis eosinophils play a pathogenic role in a subset with FD or FD and IBS overlap.^40^

Interestingly like atopic diseases, the incidence of FD is increasing, at least in Sweden, based on prospective follow-up data pre-SARS-CoV-2.^55^ In support of eosinophils playing a pathogenic role in FD, alterations in duodenal neuronal structure and function have been documented in FD versus controls, and increased small intestinal permeability has been observed; these alterations correlate with the microscopic inflammation present.^39,56,57^

Rumination syndrome characterized by effortless regurgitation is now recognized to be a relatively common DGBI that significantly overlaps with FD. In a US population-based study, the prevalence of rumination syndrome and FD was surprisingly similar (5.8% and 7.1%, respectively) and these disorders overlapped four-times more often than expected by chance.^58^ Similar prevalence results were reported in the Rome epidemiology global survey of internet panel volunteers.^59^ A Mayo Clinic study of 22 patients with confirmed rumination syndrome observed there was an increase in the mean eosinophil count among rumination syndrome versus 10 controls (26 per mm^2^ (range 16–42) versus 18 per mm^2^ (range 10–28), *p* = .006). Intraepithelial lymphocyte counts were also significantly higher in patients with rumination syndrome, which has not been seen in FD alone.^60^ A paediatric study also observed increased duodenal eosinophils in rumination syndrome but further confirmatory research is awaited.^61^

Not only does rumination overlap with FD. Gastroesophageal reflux disease is well established to occur more often than expected by chance in FD, but the reason these disorders overlap has been obscure.^62^ Following up our Swedish population over a decade, we observed a ninefold increased risk of incident symptomatic GERD in those with FD and post-prandial distress syndrome at baseline, and further among those with duodenal eosinophilia and postprandial distress syndrome a sixfold increased risk of incident GERD.^63^

These results implicating the duodenum in the pathogenesis of new-onset GERD remain to be confirmed, but both GERD and rumination may be explained by exaggerated duodeno-gastro-oesophageal reflexes in the setting of duodenal microinflammation and visceral hypersensitivity; the reflex response slows gastric emptying and increases relaxation of the lower oesophageal sphincter.^64^ Abnormal duodenal hormone release (e.g., cholecystokinin) may also play a role.^64,65^ Duodenal eosinophilia in FD is also associated with an over fourfold increased risk of anxiety over 10 years implicating inflammation in driving psychological distress in some with FD.^66^

Of interest, celiac disease and Crohn’s disease are also associated with eosinophilic infiltration in the small intestine, and eosinophils may play a role in disease pathogenesis.^67,68^ Eosinophils have also been potentially implicated in the pathogenesis of diverticular disease.^69^ In IBS increased intestinal eosinophils have not been identified in fasting patients,^70^ but in the colon in those with colonic spirochetosis and diarrhoea-predominant IBS there is eosinophil infiltration,^71^ and in IBS following food antigen exposure (e.g., wheat) increased duodenal eosinophilia acutely has been observed in more than 50% of cases by confocal microscopy.^72^

## Eosinophilic duodenitis and FD: cause or consequence?

A major subgroup of patients presenting with FD have increased duodenal eosinophils, but the eosinophil counts are usually not in the high ranges reported in non-oesophageal EGIDs. This raises the question are these eosinophils just a biomarker of disease or could they play a fundamental role in the pathogenesis? The jury remains out, but there is growing evidence even that modestly increased tissue eosinophils are not benign.^34,73^

There is increasing evidence that duodenal eosinophils are activated and degranulate in FD, potentially releasing toxic by-products that can induce tissue damage.^31,74^ In an elegant study, Wauters et al. evaluated 30 healthy volunteers, 27 FD patients who were started on a proton pump inhibitor (PPI), and 18 FD patients who ceased this drug class for the experiments. They observed microinflammation and increased duodenal permeability, but FD symptoms correlated only with eosinophils both before and during PPI therapy.^39^

PPIs are an established drug therapy in FD although only a subgroup are responders.^2,3^ Clinical studies suggest PPIs suppress duodenal eosinophils in FD and this correlates with their symptom benefit, so acid suppression may be less important.^75^ However, randomized controlled trials confirming the anti-eosinophil action of PPIs are currently lacking.

Budesonide has been trialled in FD with duodenal eosinophilia in a pilot study.^76^ Overall, the locally acting steroid was not shown to provide a symptom benefit over placebo but in those whose eosinophil counts decreased there was a significant correlation with symptom reduction.^76^ One of the problems with this small trial was it is uncertain how reliably the budesonide was released at the site of disease (proximal duodenum) which may have impaired detecting a definitive outcome.^76^

## Why are eosinophils increased in FD: what are the underlying causes of FD?

In addition to increased activated and degranulating duodenal eosinophils in FD, now an established biomarker, increased duodenal permeability and systemic immune activation have been documented in the disorder.^39,77,78^ Further, we have reported a Th2 and Th17 signature in the human duodenal mucosa in FD and identified the presence of effector and memory cells suggesting that the duodenal eosinophilia in FD is food or bacterial antigen-driven.^56^ Cytokine (e.g., TNF alpha) and neuropeptide release may account for extra-intestinal symptoms including anxiety.^50,79^

Mast cells can be increased in the duodenum in FD and may play a role in barrier disruption^39,80^ but there is at most a modest increase above normal and mast cell activation is likely variable.^32,54,56^ Further, in a maternal separation rat model, eosinophils but not mast cells were significantly increased in the gastroduodenum; this inflammation was linked to increased gastric visceral hypersensitivity, and reversed by treating the tissue eosinophilia with dexamethasone.^81^

Several pathogenic pathways may lead to the observed duodenal pathological and immune changes resulting in FD symptoms. We propose a 2 hit-hypothesis for FD.

### Infectious gastroenteritis

There is compelling evidence FD with or without IBS arises after an episode of gastroenteritis. Bacteria have often been implicated as well as giardia.^82^ Increased intestinal permeability can occur in 50% of patients after *Campylobacter jejuni* and 30% after *Salmonella* gastroenteritis.^82^ Postinfectious FD can also arise after viral infections including SARS-CoV-2 with an eightfold increased risk.^83^ Although only about 20% recall FD beginning after an attack of gastroenteritis, it is possible infection is often missed as the precipitating cause.^3^

### Microbiome

Small intestinal bacteria overgrowth is present in FD with a fourfold increased risk, implicating microbial alterations in the pathogenesis. However, breath tests are inaccurate and duodenal fluid cultures miss most bacteria.^84^ On the other hand, using qPCR, bacterial load is increased in patients with DGBIs and this was not explained by PPI use.^85^

**Fig. 2.**
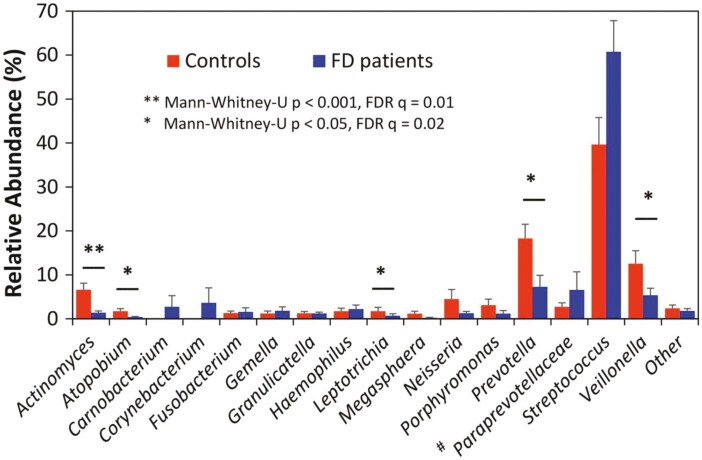
Duodenal mucosa-associated microbiome in functional dyspepsia versus controls by 16S rRNA gene sequencing. Differences in relative abundance of bacterial genera between patients with functional dyspepsia (FD) and controls were established using the Mann–Whitney *U* test, with false discovery rate (FDR) correction for multiple comparisons. The dominant phylum are Firmicutes, genus Streptococci. From Zhong L, Shanahan ER, Raj A, Koloski NA, Fletcher L, Morrison M, Walker MM, Talley NJ, and Holtmann G. “Dyspepsia and the Microbiome: Time to Focus on the Small Intestine.” *Gut* 66, no. 6 (June 2017):1168–1169. Copyright 2017 by GUT. Figure reproduced with permission of GUT in the format of a figure via Copyright Clearance Center.

We were the first to identify an increased relative abundance of *Streptococcus* spp. in the duodenum of patients with FD compared with controls, and an inverse relationship between *Streptococcus* abundance and that of the anaerobic genera *Prevotella*, *Veillonella*, and *Actinomyces*, all significantly reduced in patients with FD^86^ (fig. 2). We have confirmed these findings in larger studies,^87–89^ as have others in Japan.^90^ In exciting work, we have most recently isolated three novel *Streptococcus salivarius* strains from patients with FD, including one (named AGIRA0003)^91^ which induces an immune response and is a candidate pathobiont we are further characterizing.

Further support for the hypothesis the microbiota is involved in the pathogenesis of FD comes from studies of the non-absorbable antibiotic rifaximin. In one randomized controlled trial rifaximin was superior to placebo in terms of symptom reduction in FD,^92^ and similar results were obtained in an observational study.^93^

It is possible that specific microbial components, such as a metabolites and/or the specific degradation of digested food by the microbiota are responsible for altering the hosts homeostatic levels which may implicate them as a key driver of DGBIs. However, adequately powered studies with consistent sampling, assessment of mucosal microbial load, and -omic approaches (i.e., combination of metatranscriptomics, metabolomics, proteomics, and shotgun metagenomics) are required to address this hypothesis.

### Food antigens

An atypical non-IgE food allergy may explain why there are increased duodenal eosinophils in a subset with FD, similar to the mechanism implicated in EoE and other EGIDs.^30^

The immune system and the gut microbiota likely work together to maintain homeostasis in the GI tract when it is exposed to the diverse array of proteins consumed in food, which undergo digestion by host-produced enzymes before reaching the small intestine.^94^ Active regulation of immune responses occurs so that when the small intestine is exposed to a food antigen, it is not automatically recognized as a threat.^95^ However, some dietary proteins resist digestion in the upper GI tract, remaining intact in the duodenum. A hypersensitivity reaction can result if these proteins are recognized by antigen-presenting cells and activate immune responses.^96^ Common food-derived allergens implicated include proteins derived from wheat, milk, wheat, soy, eggs, nuts, and fish/shellfish.^97^

As wheat is the most common food trigger reported in FD and IBS, considerable research has been conducted into potential mechanisms of symptom induction, but differentiating which components influence which physiological and immune-mediated responses remains elusive.^3^ Gluten induces bloating and abdominal pain in IBS and epigastric pain and post-prandial distress in FD.^3^ Proposed immune-mediated mechanisms for induction of these symptoms could include toll-like receptor activation by gluten-derived peptides, initiating inflammatory responses,^98^ increased duodenal eosinophils promoting an inflammatory state associated with increased innervation causing visceral hypersensitivity,^99^ or gluten-derived peptides causing decreased tight junction protein integrity.^99^ Although the fructan (FODMAP) compounds in wheat are mainly considered to induce symptoms via fermentation and gas release, they may also have an immunomodulatory effect that contributes to inflammation.^100,101^

In other patients with FD, visceral hypersensitivity from microscopic inflammation to gases produced from fermentation of dietary substrates is a potential physiological mechanism for epigastric pain induction, which may explain the response to a low fermentable carbohydrate diet in FD.^102,103^

### Smoking

Combining three population-based studies, smoking was a risk factor for FD postprandial distress syndrome (but not epigastric pain syndrome), with a dose-response relationship.^104^ Smoking increases duodenal eosinophils^105^ and intestinal permeability,^106^ and alters the duodenal microbiota to a pattern more consistent with that observed in FD.^107^

### Drugs

Non-steroidal anti-inflammatory drugs (NSAIDs) including aspirin can increase intestinal permeability in DGBIs more than in healthy controls^108^ and are associated with increased FD symptoms.^109^

We have shown in a human experimental model that subjects who do not develop new onset dyspepsia on aspirin treatment had a significant increase of intestinal sensory thresholds, whereas when thresholds remained unchanged or decreased subjects were more likely to develop symptoms.^110^ Exposure to antibiotics is also associated with an increased risk of FD, consistent with the concept altering the microbiome increases risk.^111^

### Stress

Anxiety is strongly associated with FD and may be driven in some by an exaggerated stress response and release of stress hormones.^112^ In healthy humans, stress can increase small intestinal permeability.^113^

We have shown in FD there is reduced duodenal corticotrophin releasing hormone (CRH)-receptor 2, compared to controls, and this correlated with processes critical for maintaining goblet cell homeostasis (namely, reduced NLRP6 expression and autophagy functions). We then confirmed duodenal goblet cell numbers and mucin exocytosis to be reduced in patients with FD.^114^

### Genetics

There is emerging that evidence genes play a role in FD and other DGBIs.^115^ For example, FD is associated with the GNβ3 825C>T CC genotype but this gene was not linked with treatment response to antidepressants in FD.^116–118^ However, it remains unknown if a genetic predisposition is linked to the risk of increased small intestinal permeability, microinflammation, or immune activation in FD.

### Two-hit hypothesis

**Fig. 3.**
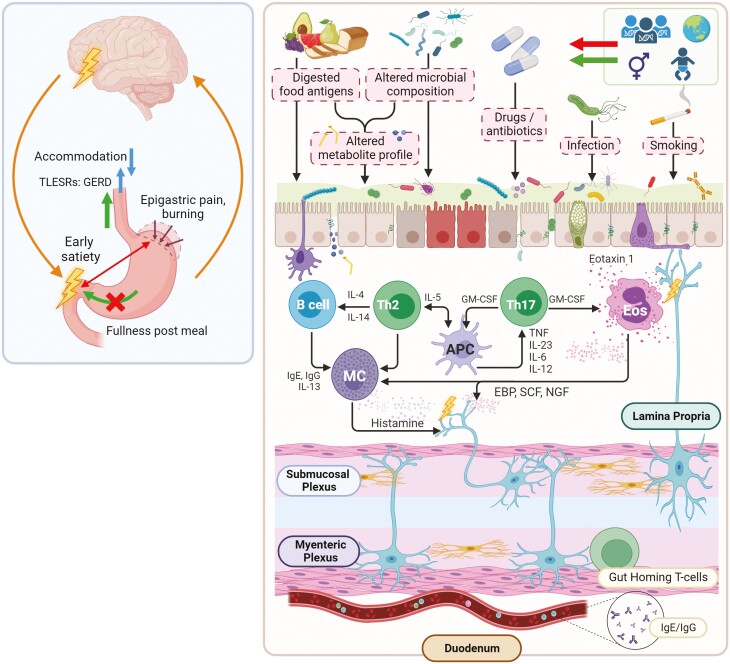
Pathogenesis of eosinophilic functional dyspepsia (EoFD): two-hit hypothesis. 1. The duodenal barrier is breached after infection, exposure to cigarettes or drugs, or in acute stress. 2. In a genetically primed individual, antigen presentation to the small intestinal mucosa then occurs (food macromolecules and/or microbial antigens). 3. Activation of eosinophils and mast cells through an immune cascade. 4. Local nerve sensitization and systemic immune activation leading to symptomatology. 5. Maintenance of a low-grade state of inflammation through bidirectional gut–brain and brain–gut pathways resulting in fluctuating symptoms [Image created in BioRender].

We propose an environmental factor that induces intestinal damage is the initiating event in FD.^3,73,119^ This could be an acute infection that clears, or exposure to NSAIDs or smoking, or a major life stressor. However, this insult alone is not enough to result in pathological immune activation in the small intestine unless there is a change in microbiota that persists. In a genetically predisposed individual, increased permeability then exposes food and microbial antigens that induce activation of the intestinal immune system; bacterial digestion could further expose food epitopes to the immune system. Once the intestinal immune system is activated, eosinophils and mast cells are likely recruited and become activated and degranulate, leading to further permeability damage (and failure of the mucosa to repair), as well as structural and functional alterations of submucosal nerves resulting in motor and sensory dysfunction, and abnormal reflex responses that manifest as gastroduodenal and in some oesophageal symptoms. Cytokine and neuropeptide release may lead to extraintestinal symptoms such as fatigue and sleep disturbances, and exacerbate or initiate anxiety (Fig. 3). Maintenance of a low-grade state of inflammation through bidirectional gut–brain and brain–gut pathways with intestinal immune activation may explain why the disease is chronic but symptoms often fluctuate, and why FD is more prevalent in females.

## Conclusions

There is now reasonably convincing evidence that activated duodenal eosinophils are increased in FD, with a meta-analysis of 22 case–control studies demonstrating eosinophil degranulation with no heterogeneity (odds ratio 3.78; 95% CI, 4.48–6.76).^54^ Counting eosinophils is *not* standardized and likely explains the diagnostic heterogeneity in the literature (including clinicians failing to take a sufficient number of biopsies, and pathologists not counting eosinophils as is routine when searching for eosinophilic esophagitis, which may explain why disease is often missed). A provisional threshold for a definitely abnormal duodenal eosinophil count is ≥30/high power field in 3–5 hpf but even lower counts (>5/higher power field in 5 hpf) may be pathological. To identify disease, it is useful to consider eosinophil distribution (e.g., excess clusters) and degranulation (thus there may be few intact eosinophils because of eosinophil traps). US data on over 500 patients suggest a significant minority with a clinical diagnosis of IBS, GERD or FD and moderate-severe refractory to treatment symptoms may have eosinophilic duodenitis. Notably, eosinophilic duodenitis is associated with neural structural and functional changes and increased small intestinal permeability. There is emerging evidence eosinophilic duodenitis may occur following infection, dysbiosis, food antigen exposure, smoking, drugs, or acute stress. PPI therapy is efficacious and first line for FD, but any benefit might be because of the drugs anti-eosinophil action. Duodenal microinflammation and immune activation could explain the GI and non-GI manifestations of FD, with diagnostic and treatment implications.

## Data Availability

There are no data associated with this manuscript.
